# A novel mutation in MBTPS2 causes ichthyosis follicularis, alopecia, and photophobia syndrome

**DOI:** 10.1002/mgg3.812

**Published:** 2019-06-18

**Authors:** Yanyun Jiang, Hongzhong Jin, Yueping Zeng

**Affiliations:** ^1^ Department of Dermatology Peking Union Medical College Hospital, Chinese Academy of Medical Sciences and Peking Union Medical College Beijing China

**Keywords:** alopecia, ichthyosis follicularis, MBTPS2, photophobia

## Abstract

**Background:**

The ichthyosis follicularis, alopecia, and photophobia (IFAP) syndrome is a rare X‐linked genodermatosis characterized by noninflammatory spiny follicular hyperkeratosis, severe photophobia, and non‐scarring alopecia with variable severities. IFAP syndrome results from mutations in the gene encoding the membrane‐bound transcription factor peptidase, site 2 (*MBTPS2*).

**Methods:**

We present an 11‐year‐old male with typical clinical features of IFAP syndrome, including diffuse follicular hyperkeratosis, alopecia, photophobia, psoriasiform plaques, short statue, nail dystrophy, mental retardation, and seizures.

**Results:**

A novel missense mutation (NM_015884.4: c.1298T > C; NP_056968.1: p. L433P) in the membrane‐bound transcription factor peptidase, site 2 gene (*MBTPS2*) was identified in our patient. The heterozygous *MBTPS2* mutation was identified in his mother but not his father.

**Conclusion:**

This study demonstrated a novel *MBTPS2* mutation in a patient with IFAP syndrome and thus expands the known *MBPTS2* molecular repertoire.

## 1 INTRODUCTION

Ichthyosis follicularis, alopecia, and photophobia syndrome (IFAP, OMIM# 308205) is a rare X‐linked genodermatosis characterized clinically by ichthyosis follicularis, alopecia, and photophobia of varying degree (Megarbane & Megarbane, 2011). The disease was first recognized as a distinct entity by Macleod in 1909 (MacLeod, 1909). Recently, the genetic basis of IFAP syndrome has been confirmed to be associated with mutations of the *MBTPS2* (membrane‐bound transcription factor protease, site 2) (Oeffner et al., 2009). To date, about 60 cases with IFAP syndrome have been reported worldwide. Herein, we reported an IFAP syndrome case with a novel mutation in the *MBTPS2* gene.

## 2 CASE REPORT

An 11‐year‐old boy was presented to our dermatology department for evaluation of diffuse follicular hyperkeratosis, alopecia, and photophobia. At the age of 3 months old, complete non‐scarring alopecia including the lack of eyebrows and lashes was noted. Hyperpigmented scales with follicular hyperkeratosis appeared on his scalp, face and buttocks, which later progressed to the whole body. Hyperkeratotic plaques developed around the regions of his elbows, knees, and buttocks. There were prominent dystrophic and deformed nails. The patient was suggested to use moisturizers for his involved skin, but no improvement was reported. He had photophobia without structural abnormalities of the eyes at the age of 1 year. Between the age 1 to 7, he suffered from several epilepsy attacks and since then anticonvulsants were administered. During the last 4 years, he did not suffer from any seizures. He had mild mental retardation and did not cope well in school for his age. He had normal hearing, sweating secretion, and dentition development. He was the son of his nonconsanguineous parents born at term weighing 3.2 kg. The pregnancy history of his mother was unremarkable. His parents and two older sisters were healthy with none of the similar manifestations, whereas his grandmother had the history of dry skin and lamellar desquamation on the lower limbs since birth.

**Figure 1:**
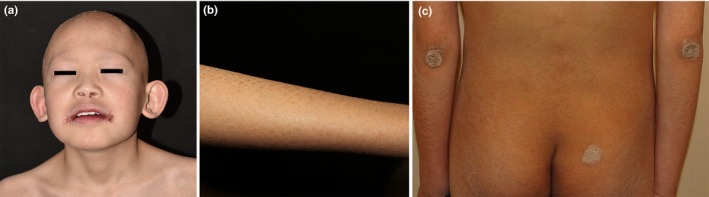
The clinical findings in IFAP. (a) Absence of hair, eyelashes, and eyebrows and chronic angular cheilitis around the mouth. (b) Generalized dry skin with widespread follicular papules and flaky scales on the arm. (c) Hyperkeratotic plaques on the elbows and buttock

**Figure 2:**
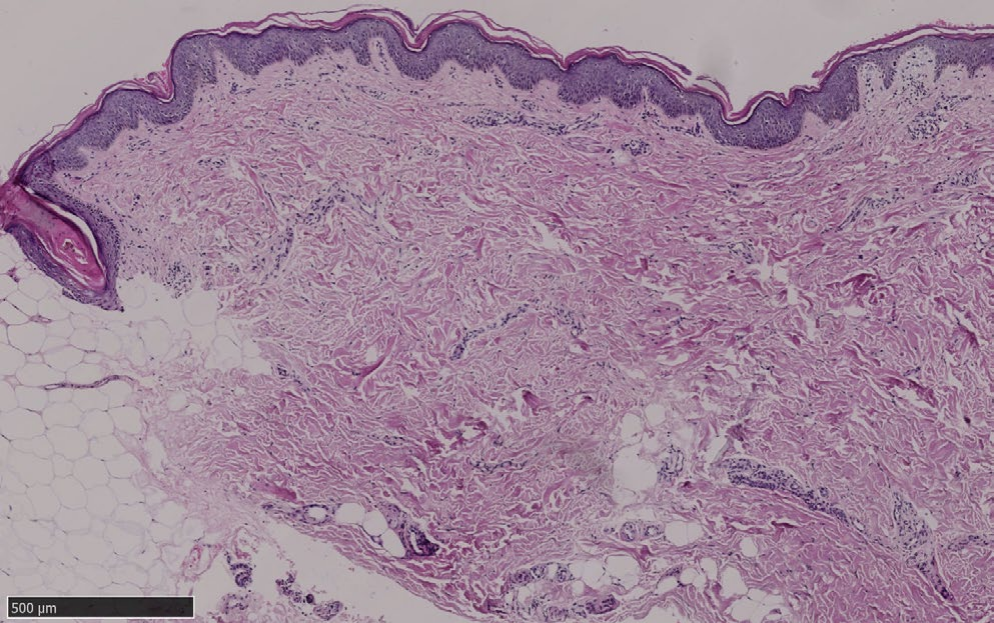
Histopathological examination of the lesion from the right limb showed hyperkeratosis, focal parakeratosis and follicular plugging of the epidermis, absence of sebaceous glands and mild perivascular inflammatory infiltration in dermis (hematoxylin and eosin staining, bar = 500 μm)

On admission, he was found to have short stature (132 cm) and low body weight (27 kg). Visual acuity was 0.25 in the left eye and 0.15 in the right eye. Ophthalmologic examination revealed conjunctival congestion and photophobia while cornea and ocular fundus were normal. He had slightly prominent ears. Dermatological examination revealed complete absence of hair, eyelashes, and eyebrows as well as chronic angular cheilitis around the mouth (Figure 1a). Generalized dry skin with widespread follicular papules involved his scalp, neck, trunk and extremities, giving a thorn‐like sensation at palpation. Flaky scales developed on the extensor extremities (Figure 1b). There were hyperkeratotic psoriasis‐like lesions on the elbows, knees, and buttocks and dark brown scales on the upper and lower limbs (Figure 1c). Histopathologic examination of a skin biopsy from the right limb showed hyperkeratosis, focal parakeratosis, and follicular plugging of the epidermis, absence of sebaceous glands and mild perivascular inflammatory infiltration in the dermis (Figure 2).

## 3 ETHICAL COMPLIANCE

The study was approved by the clinical research ethics committee of Peking Union Medical College Hospital, Chinese Academy of Medical Sciences and Peking Union Medical College. Written informed consents were obtained from the patient and his parents.

## 4 MOLECULAR GENETIC STUDIES

Peripheral blood was sampled from the patient and his parents for genomic DNA extraction using a commercial kit (Qiagen FlexiGene DNA kit) according to the manufacturer's instruction. Genetic investigations for their daughters and grandmother were not available. Genomic DNA sample was fragmented to construct DNA library. Then the DNA library was amplified and purified by PCR in accordance with the manufacturer's instructions. Single‐read sequencing was performed by NextSeq500 (illumina). Then Align analysis, SNP analysis, and DIP analysis were conducted to obtain information of mutation sites from targeted region. At last, protein damage analysis was conducted to qualitatively predict the probability of the results by PolyPhen‐2.2.2. In addition, variants were confirmed by Sanger sequencing in final.

**Figure 3:**
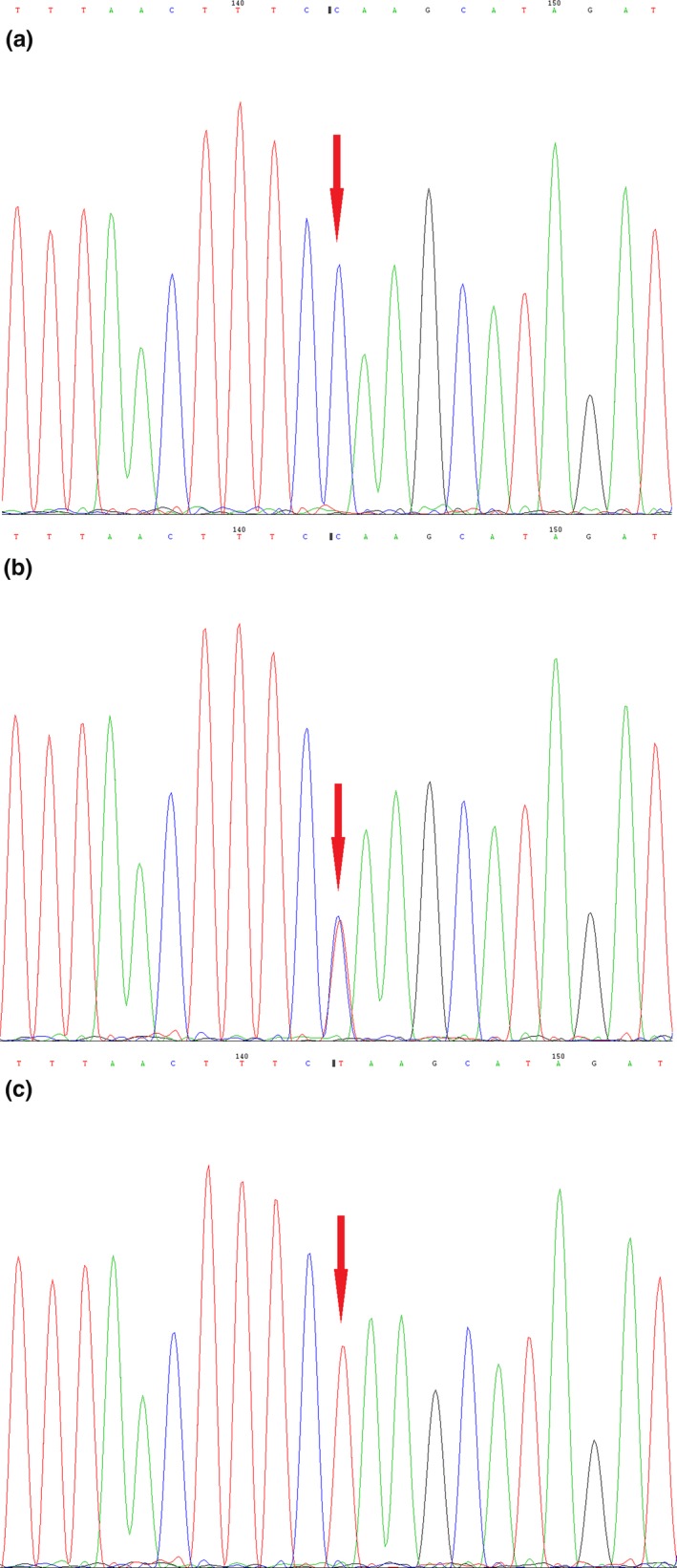
Genomic DNA sequencing of the proband and his parents. (a) Mutation of c.1298T > C is hemizygous in the proband as there is only one X chromosome. (b) The same mutation in heterozygous form was identified in his mother. (c) The sequence of the proband's father was normal

Exome sequencing revealed a mutation c.1298T > C (p. L433P) of *MBTPS2* in exon 10 of the X phenotype of IFAP chromosome. The heterozygous *MBTPS2* mutation was identified in his mother but not his father (Figure 3). Based on the history, clinical examination, histological findings, and *MBTPS2* gene mutation, the patient was diagnosed as IFAP syndrome. He was started on acitretin 10 mg per day and still under follow‐up.

## 5 DISCUSSION

The IFAP syndrome is a rare disease characterized by typical presence of ichthyosis follicularis, alopecia and photophobia. Ichthyosis follicularis is the most common cutaneous manifestation presenting as noninflammatory follicular keratotic papules, which mainly involve the scalp and extensor extremities and give the sandpaper texture. Other cutaneous findings include hyperkeratotic psoriasiform plaques, lamellar scaling, angular cheilitis, periungual inflammation, and dystrophic nails. Congenital non‐cicatricial alopecia involving the scalp, eyebrows, and eyelashes is another distinct finding. Most of the cases have complete body hair loss, while some cases have thin and sparse hair (Khandpur, Bhat, & Ramam, 2005). Photophobia is an essential feature for the diagnosis of IFAP, which may exist since birth or later in childhood. It is associated with superficial corneal ulceration and vascularization that leads to vision loss (Traboulsi, Waked, Megarbane, & Megarbane, 2004). Other ocular manifestations include corneal erosions and scarring, chronic tearing, atopic keratoconjunctivitis, nystagmus, and myopia. It is usually normal for the anterior chamber, lens and ocular fundus. Some patients have additional manifestations including brain anomalies, intellectual disability, ectodermal dysplasia, skeletal deformities, ear or eye anomalies, and kidney dysplasia, Hirschsprung's disease and cleft palate or cryptorchidism (BRESHECK syndrome), which is considered as the severe phenotype of IFAP syndrome(Corujeira et al., 2013). Skin histopathology is nonspecific and consists of follicular plugging, absent sebaceous glands and normal sweat glands (Kamo et al., 2011).

The genetic inheritance pattern in IFAP had previously been assumed to be X‐linked, but as a few female patients were reported, an alternative autosomal dominant mode of transmission had been proposed (Megarbane & Megarbane, 2011). In 2009, mutations in *MBTPS2* gene located in Xp22.11‐p22.13 have been reported to cause IFAP syndrome (Oeffner et al., 2009). MBTPS2, the protein encoded by the *MBTPS2* gene, is a membrane‐embedded zinc metalloprotease essential for sterol control of transcription and endoplasmic reticulum stress response. Eight transmembrane domains (TM 1 to TM 8, N‐terminal to C‐terminal) span the endoplasmic reticulum membrane. Mutations in *MBTPS2* were also identified as genetic basis for IFAP syndrome with BRESHECK syndrome, keratosis follicularis spinulosa decalvans syndrome (KFSD; OMIM# 308800) and an X‐linked form of Olmsted syndrome (OMIM# 300918) (Bornholdt et al., 2013). Previous studies showed that MBTPS2 has a HEIGH motif and an LDG motif, which help to coordinate the zinc atom in the active site of the enzyme. Mutations close to hydrophobic, presumably intramembranous, domain encompassing the LDG motif at the active site, are more detrimental to development than an amino acid substitution in the N‐terminal part of the protein (Oeffner et al., 2009). For instance, the mutation of p.R429H, p.F475S, p.L476S and p.D477V are close to the LDG motif, which clinically result in severe IFAP phenotypes (Bornholdt et al., 2013; Oeffner et al., 2009). Besides that, Dorothea Bornholdt et al demonstrated that the site of the mutation in *MBTPS2* is associated with clinical presentations (Bornholdt et al., 2013). The mutation p.M87I within the second TM domain is related with a mild form of IFAP syndrome, while the mutations of p.F229S, p.W226L and p.H227L within TM5 are associated with pronounced IFAP triad. The mutation of p.R429H has been independently described in IFAP syndrome with BRESHECK syndrome. The mutation of p.N508S was identified in three unrelated families as a cause for KFSD. The mutation of p.F464S was found in a patient with the X‐linked form of Olmsted Syndrome and the intronic mutation c.671‐9T > G of *MBTPS2* was found in IFAP patient with Olmsted syndrome‐like features(Haghighi et al., 2013; Wang et al., 2014). To date, IFAP syndrome has been found to affect 19 ethnic populations associated with 20 mutations in *MBTPS2* (Table 1). In this case, we detected a novel missense mutation, which caused a replacement from leucine to proline at amino acid residue 433 in the MBTPS2 protein (p. L433P). The variant results in the substitution of highly conserved amino acid residues located within TM7. The mutation p. L433P occurred adjacent to the previously described exchanges p. R429H within TM7, and these two mutations may present similar clinical features. Previous studies reported that the mutation p. R429H was associated with severe IFAP phenotype, which showed additional features such as mental retardation, seizures, visceral and skeletal anomalies (Bornholdt et al., 2013). We reported that this mutation featured with short statue, nail dystrophy, mental retardation, seizures in addition to IFAP triad.

**Table 1:** The list of *MBTPS2* mutations that have been identified to be associated with IFAP syndrome

Mutation	Protein variant	Published year	Origin	Sex	Number of subjects
c.680A > T	p.H227L	2009 (Oeffner et al., 2009)	Germany	Male	1
				Female	3
c.261G>A	p.M87I	2009 (Oeffner et al., 2009)	Australia	Male	3
				Female	5
		2013 (Bornholdt et al., 2013)	Sweden	Male	1
			United Kingdom	Male	1
c.1286G>A	p.R429H	2009 (Oeffner et al., 2009)	Germany	Female	5
		2011 (Nakayama et al., 2011)	Japan	Male	1
		2013 (Bornholdt et al., 2013)	Canada	Male	1
		2013 (Bornholdt et al., 2013)	France	Male	1
				Female	1
c.667G>T	p.W226L	2009 (Oeffner et al., 2009)	Spain	Male	1
c.1424T>C	p.F475S	2009 (Oeffner et al., 2009)	Argentina	Male	1
		2017 (Nemer et al., 2017)	Lebanon	Male	2
		2013 (Bornholdt et al., 2013)	Lebanon	Male	2
c.225‐6T>A		2009 (Oeffner et al., 2009)	Algeria	Male	2
				Female	1
		2011 (Oeffner et al., 2011)	North Africa	Male	1
c.1523A>G	p.N508S	2010 (Ding, Wang, Qiao, Mao, & Cai, 2010)	China	Male	1
		2013 (Bornholdt et al., 2013)	Sweden	Male	1
c.1433C＞A	p.A478D	2011 (Tang, Liang, Wang, Yu, & Yao, 2011)	China	Male	1
c.671‐9T>G		2011 (Oeffner et al., 2011)	Canada	Male	1
			Ashkenazi	Male	1
		2014 (Wang et al., 2014)	China	Male	1
c.1001G>A	p.C334Y	2012 (Pietrzak et al., 2012)	Poland	Male	1
c.71T>C	p.L24P	2013 (Izumi, Wilkens, Treat, Pride, & Krantz, 2013)	USA	Male	1
c.774C>G	p.I258M	2013 (Bornholdt et al., 2013)	USA	Male	1
c.758G>C	p.G253A	2013 (Bornholdt et al., 2013)	Germany	Male	1
				Female	1
c.686T>C	p.F229S	2013 (Bornholdt et al., 2013)	France	Male	1
				Female	3
c.1427T>C	p.L476S	2013 (Bornholdt et al., 2013)	Germany	Male	1
				Female	2
c.1430A>T	p.D477V	2013 (Bornholdt et al., 2013)	Algeria	Male	1
c.1499G>A	p.G500D	2013 (Bornholdt et al., 2013)	The Netherlands	Male	1
				Female	1
			Syria	Male	2
c.1538T>C	p.L513P	2013 (Bornholdt et al., 2013)	Sri Lanka	Male	1
c.1523A>C	p.N508T	2015 (Fong et al., 2015)	United Kingdom	Male	1
c.1360G>C	p.A454P	2015 (Araujo, Goncalves‐Rocha, Resende, Vieira, & Brito, 2015)	Canada	Male	1

Differential diagnoses should include other conditions in which generalized ichthyosis and alopecia are the main features. These conditions include KFSD syndrome, hereditary mucoepithelial dysplasia (HMD; OMOM# 158310), and the keratosis, ichthyosis, and deafness syndrome (KID syndrome; OMIM# 242150). IFAP syndrome is not treatable. Oral acitretin at a dose of 0.3 to 1 mg/kg/day had been used with improvement in cutaneous features but no changes for photophobia (Megarbane & Megarbane, 2011).

In conclusion, we reported a boy with clinical and histological features of IFAP syndrome. A novel missense mutation c.1298T > C (p. L433P) in MBTPS2 gene was detected, which added new genetic information to this condition.

## CONFLICTS OF INTERESTS

The authors report no conflict of interest.
